# Supplementary material for: The Tryptophan Catabolite or Kynurenine Pathway in a Major Depressive Episode with Melancholia, Psychotic Features and Suicidal Behaviors: A Systematic Review and Meta-Analysis
Source: Cells. 2022 Oct 2;11(19):3112. doi: 10.3390/cells11193112 (PMC9563030; doi:10.3390/cells11193112)
Supplement: Supplementary file 1 [file cells-11-03112-s001.zip › cells-1898916-supplementary.pdf]

The tryptophan catabolite or kynurenine pathway in a major depressive episode with melancholia, psychotic features and suicidal behaviors; a systematic review and meta-analysis.

SHORT TITLE: Kynurenine pathway in affective disorders

Abbas F. Almulla, Ph.D.[a,b] , Yanin Thipakorn, M.D., Ph.D.[a], Asara Vasupanrajit, MS.c[a], , Chavit Tunvirachaisakul, M.D., Ph.D.[a, c], Gregory Oxenkrug, M.D., Ph.D.[d], Hussein K. Al-Hakeim, Ph.D.[e], Michael Maes, M.D., Ph.D.[a,f,g]

[a] Department of Psychiatry, Faculty of Medicine, Chulalongkorn University, Bangkok, Thailand.

[b] Medical Laboratory Technology Department, College of Medical Technology, The Islamic University, Najaf, Iraq.

[C] Cognitive Impairment and Dementia Research Unit, Faculty of Medicine, Chulalongkorn University, Bangkok, Thailand.

[d] Department of Psychiatry, Tufts University School of Medicine and Tufts Medical Center, Boston, MA 02111, USA.

[e] Department of Chemistry, College of Science, University of Kufa, Kufa, Iraq.

[f] Department of Psychiatry, Medical University of Plovdiv, Plovdiv, Bulgaria.

[g] Deakin University, IMPACT - the Institute for Mental and Physical Health and Clinical Translation, School of Medicine, Barwon Health, Geelong, Australia.

Table S1. Search sentences and terms were used in each database

Database Name	Search Sentence	No. of Articles
PubMed/Medline	((((((((((TRYCATs and Suicide)* OR (kynurenine pathway and Suicide)) OR (Tryptophan Catabolites and Suicide)) OR (L-TRP and Suicide)) OR (L-KYN and Suicide)) OR (KYNA and Suicide)) OR (ANA and Suicide)) OR (3-HK and Suicide)) OR (XA and Suicide)) OR (3-HA and Suicide)) OR (QA and Suicide)) OR (PA and Suicide)	**7714**
	(((((((((Melancholia and TRYCATs)* OR (Melancholia and Tryptophan)) OR (melancholia and tryptophan cata)) OR (Melancholia and TRYCATs)) OR (Melancholia and TRP)) OR (Melancholia and KYN)) OR (Melancholia and kynurenine)) OR (Melancholia and Kynurenine pathway)) OR (Melancholia and IDO)) OR (Melancholia and TDO)	**1283**
	(((((((((Psychotic depression and TRYCATs)* OR (Psychotic depression and Tryptophan)) OR (Psychotic depression and tryptophan cata)) OR (Psychotic depression and TRYCATs)) OR (Psychotic depression and TRP)) OR (Psychotic depression and KYN)) OR (Psychotic depression and kynurenine)) OR (Psychotic depression and IDO)) OR (Psychotic depression and TDO)	**81**
Google Scholar	(((((Melancholia* and IDO activation) OR (TDO activation)) OR (Kynurenine pathway)) OR (KMO activation)) OR (Tryptophan degradation)) AND (Decreased Tryptophan)	**499**
	(((((Psychotic depression * and IDO activation) OR (TDO activation)) OR (Kynurenine pathway)) OR (KMO activation)) OR (Tryptophan degradation)) AND (Decreased Tryptophan)	**1250**
SciFinder	Suicide and kynurenine pathway, Suicide and tryptophan catabolism pathway, Suicide and TRYCATs, Suicide and kynurenine, Suicide and tryptophan, Suicide kynurenic acid, Suicide and kynurenine pathway	**9**

	Melancholia and kynurenine pathway, Melancholia and tryptophan catabolism pathway, Melancholia and TRYCATs, Melancholia and kynurenine, Melancholia and tryptophan, Melancholia and kynurenic acid, Psychotic depression and kynurenine pathway	**25**

Table S2 . Immune cofounder's scale (ICS); adapted from Andrés-Rodríguez, et al., 2019

**Methodological quality of the study**		
**1**	Study sample ≥ 128 participants including patients and controls (1= Yes, 0 = No)	
**2**	Did the study control the results for potential confounders (e.g., age, BMI, gender, race)? (1= Yes, 0 = No)	
**3**	Were participants with affective disorders and controls age- and-gender-matched or was there a statistical control? (1= Yes, 0 = No)	
**4**	Was the time of sample collection specified (e.g., morning vs. evening)? (1= Yes, 0 = No)	
**5**	Were participants with affective disorders free of immunomodulatory drugs including anti-cytokines, glucocorticoids, immunoglobulins, and immunosuppressants, or was there a medication washout period, or was drug intake statistically controlled for? (1= Yes, 0 = No)	
**6**	Were participants with affective disorders free of antidepressants and mood stabilizers or were the data statistically controlled for? (1= Yes, 0 = No)	
**7**	Reporting either the manufacturer of the test or detection limit and coefficients of variation (1= Yes, 0 = No)	
**8**	Reporting how data under detection limit were handled (1 = Yes, 0 = No)	
**9**	Reporting % of the sample under detection limit (1=Yes, 0= No)	
**10**	Reporting blood fraction (serum, plasma, culture supernatant or whole blood) (1= Yes, 0 = No)	
		**Total quality score (10 points)**
**Biomarker confounders red points**		

The red points should not be given if the item is statistically controlled for		
**1**	3 red points for comorbid illnesses such as autoimmune disorders & other immune disorders including rheumatoid arthritis, psoriasis, inflammatory bowel disease, chronic obstructive pulmonary disease, multiple sclerosis	
**2**	3 red points for use of recreational drugs such as methamphetamine or opioids	
**3**	2 red points when groups were not matched for age	
**4**	2 red points when groups were not matched for sex	
**5**	2 red points for medication use as for example immunomodulators	
**6**	2 red points for early traumatic life events	
**7**	2 red points for shift work and primary sleep disorders	
**8**	1.5 red points for use of antipsychotics	
**9**	1 red point for more common systemic immune disorders including diabetes type 1/2, essential hypertension, metabolic syndrome	
**10**	1 red point for not fasting (8 hours before blood extraction)	
**11**	1 red point for use of omega-3 and antioxidant supplements	
**12**	1 red point when data were not controlled for body mass index	
**13**	1 red point when data were not controlled for physical activity or sedentary life	
**14**	1 red point when data were not controlled for smoking	

**15**	1 red point for use of oral contraceptives or NSAIDs	
**16**	0.5 red points when data were not controlled for ethnicity in countries such as US, Brazil	
**17**	0.5 red points when data were not controlled for seasonality	
**18**	0.5 red points when data were not controlled for diurnal variation (8-10 a.m. versus all other time points)	
	**Total red point score (26 points)**	

Table S3. PRISMA checklist

Section/topic	#	Checklist item	Reported on page #
**TITLE**			
Title	1	Identify the report as a systematic review, meta-analysis, or both.	1
**ABSTRACT**			
Structured summary	2	Provide a structured summary including, as applicable: background; objectives; data sources; study eligibility criteria, participants, and interventions; study appraisal and synthesis methods; results; limitations; conclusions and implications of key findings; systematic review registration number.	3
**INTRODUCTION**			
Rationale	3	Describe the rationale for the review in the context of what is already known.	5-7
Objectives	4	Provide an explicit statement of questions being addressed with reference to participants, interventions, comparisons, outcomes, and study design (PICOS).	8
**METHODS**			
Protocol and registration	5	Indicate if a review protocol exists, if and where it can be accessed (e.g., Web address), and, if available, provide registration information including registration number.	9
Eligibility criteria	6	Specify study characteristics (e.g., PICOS, length of follow-up) and report characteristics (e.g., years considered, language, publication status) used as criteria for eligibility, giving rationale.	10
Information sources	7	Describe all information sources (e.g., databases with dates of coverage, contact with study authors to identify additional studies) in the search and date last searched.	11
Search	8	Present full electronic search strategy for at least one database, including any limits used, such that it could be repeated.	9-10, ESF, table 1
Study selection	9	State the process for selecting studies (i.e., screening, eligibility, included in systematic review, and, if applicable, included in the meta-analysis).	10-11, Figure 2

Data collection process	10	Describe method of data extraction from reports (e.g., piloted forms, independently, in duplicate) and any processes for obtaining and confirming data from investigators.	11
Data items	11	List and define all variables for which data were sought (e.g., PICOS, funding sources) and any assumptions and simplifications made.	11
Risk of bias in individual studies	12	Describe methods used for assessing risk of bias of individual studies (including specification of whether this was done at the study or outcome level), and how this information is to be used in any data synthesis.	12-13
Summary measures	13	State the principal summary measures (e.g., risk ratio, difference in means).	12-13
Synthesis of results	14	Describe the methods of handling data and combining results of studies, if done, including measures of consistency (e.g., I^2) for each meta-analysis.	12-13
Risk of bias across studies	15	Specify any assessment of risk of bias that may affect the cumulative evidence (e.g., publication bias, selective reporting within studies).	12-13
Additional analyses	16	Describe methods of additional analyses (e.g., sensitivity or subgroup analyses, meta-regression), if done, indicating which were pre-specified.	12-13
**RESULTS**			
Study selection	17	Give numbers of studies screened, assessed for eligibility, and included in the review, with reasons for exclusions at each stage, ideally with a flow diagram.	14
Study characteristics	18	For each study, present characteristics for which data were extracted (e.g., study size, PICOS, follow-up period) and provide the citations.	15, ESF, table 4
Risk of bias within studies	19	Present data on risk of bias of each study and, if available, any outcome level assessment (see item 12).	Table 3
Results of individual studies	20	For all outcomes considered (benefits or harms), present, for each study: (a) simple summary data for each intervention group (b) effect estimates and confidence intervals, ideally with a forest plot.	Table 1
Synthesis of results	21	Present results of each meta-analysis done, including confidence intervals and measures of consistency.	Table 2
Risk of bias across studies	22	Present results of any assessment of risk of bias across studies (see Item 15).	Table 3
Additional analysis	23	Give results of additional analyses, if done (e.g., sensitivity or subgroup analyses, meta-regression [see Item 16]).	18, ESF, table 5

**DISCUSSION**			
Summary of evidence	24	Summarize the main findings including the strength of evidence for each main outcome; consider their relevance to key groups (e.g., healthcare providers, users, and policy makers).	19-26
Limitations	25	Discuss limitations at study and outcome level (e.g., risk of bias), and at review-level (e.g., incomplete retrieval of identified research, reporting bias).	26
Conclusions	26	Provide a general interpretation of the results in the context of other evidence, and implications for future research.	26
**FUNDING**			
Funding	27	Describe sources of funding for the systematic review and other support (e.g., supply of data); role of funders for the systematic review.	27

Table S4. Characteristics of the studies included in the systematic reviews and meta-analysis

NO	Authors, years	Setting	Type of case	Type of Control	Sample Size			Age		Assessed Biomarkers	Specimen	Method	Quality score	Red point score	Findnigs
					Cases M/F	Control M/F	Total M/F	Case-Mean (SD)	Control-Mean(SD)						
1	Aarsland, Leskauskaite et al. 2019	Norway	MDD-Psychotic feature	Healthy Control	27 12/15	14 6/8	41 18/23	45,3(10,7)	-	TRP,KYN, KA,3HK, XA, AA, 3HA,QA,PA	Serum	LCMS	5,75	13,5	TRP*,KYN$^{\#}$, KA*,3HK*, XA*, AA*, 3HA*,QA$^{\#}$,PA*
2	Achtyes, Keaton et al. 2020	USA	Post partum depression-Suicidal	Healthy Control	87 0/87	60 0/60	147 0/147	26.7(5.2)	28(6.3)	TRP, KYN, KA,QA	Plasma	HPLC-UV	5,75	14	TRP*, KYN*, KA*,QA*
3	van den Ameele, van Nuijs et al. 2020	Belgium	BD-Depression-Psychotic feature	Healthy Control	35 11/24	35 16/29	70 27/53	43.7(9.7)	42.7(11.6)	TRP, KYN, 3HK,QA, KA	Plasma	UPLC-MS/MS	7,75	10	TRP*, KYN*, 3HK*,QA*, KA*
			BD-hypomania-Psychotic feature	Healthy Control	32 15/17	35 16/29	67 31/46	42.4(12.7)	42.7(11.6)	TRP, KYN, 3HK,QA, KA	Plasma	UPLC-MS/MS			TRP*, KYN*, 3HK*,QA*, KA*
4	Anderson, Parry-Billings et al. 1990	UK	MDD-Melancholia	Healthy Control	32 15/16	31 15/16	62 30/32	34.4(8.5)	33.9(7.7)	TRP	Plasma	Fluorometry	4	12,5	TRP*
5	Bay-Richter, Linderholm et al. 2015	Sweden	Affective disorders-Suicidal	Healthy Control	30 9/21	36 29/7	66 38/28	40(8,7)	29,7(7,3)	QA,KA	CSF	GCMS,HPLC	3	18,5	QA$^{\#}$,KA*
6	Bradley, Case et al. 2015	USA	MDD-Non suicidal	Healthy Control	30 18/12	22 9/13	52 27/25	15.2(1.9)	15.9(2.6)	TRP. KYN, 3HA	Plasma	HPLC	4,25	16	TRP$^{\#}$. KYN*, 3HA$^{\#}$
			MDD-suicidal	Healthy Control	20 5/15	22 9/13	42 14/28	16.8(1.8)	15.9(2.6)	TRP. KYN, 3HA	Plasma	HPLC			TRP*. KYN*, 3HA*
7	Brundin, Sellgren et al. 2016	Sweden	MDD-Suicidal	Healthy Control	22 -	36 -	58 -	42.1(13.6)	32.5(11.3)	PA	CSF	GCMS	5,5	18	PA*
			Dysthymia	Healthy Control	4 -	36 -	40 -	44.3(23.3)	32.5(11.3)	PA	CSF	GCMS			PA*
			Depression NOS	Healthy Control	9 -	36 -	45 -	30.8(6.6)	32.5(11.3)	PA	CSF	GCMS			PA*
			MDD-Suicidal	Healthy Control	15 -	29 -	44 -	43.1(13.5)	40.6(11.5)	PA, QA	Plasma	GCMS			PA*, QA$^{\#}$
			Dysthymia	Healthy Control	3 -	29 -	32 -	43.0(18.9)	40.6(11.5)	PA, QA	Plasma	GCMS			PA*, QA*
			BD	Healthy Control	21 -	29 -	50 -	39.2(12.4)	40.6(11.5)	PA, QA	Plasma	GCMS			PA*, QA$^{\#}$
			Depression UNS	Healthy Control	4 -	29 -	33 -	28.1(8.5)	40.6(11.5)	PA, QA	Plasma	GCMS			PA*, QA#

8	Busse, Busse et al. 2015	Germany	Depression-Suicidal	Healthy Control	6 2/4	10 5/5	16 7/9	47(10)	56(6)	QA	Brain Tissue	-	2,75	16	QA*
9	Clark, Pocivavsek et al. 2016	USA	Depression-Suicidal	Healthy Control	45 30/15	36 27/9	91 57/24	43.3(13.6)	42.1(14.3)	TRP,KYN, KA,3-HK, QA	Brain Tissue	HPLC,GC/MS	2,75	17,5	TRP*,KYN*, KA*,3-HK*, QA*
10	Cowen, Parry-Billings et al. 1989	UK	MDD-Melancholia	Healthy Control	12 6/6	12 6/6	24 12/12	36,5 (26-55)	36,2 (28-54)	TRP	Plasma	Fluorometry	6,75	11	TRP*
11	Dahl, Andreassen et al. 2015	Norway	MDD-Melancholia	Healthy Control	50 12/38	34 15/19	84 27/57	40(12)	38,3(13,9)	TRP,KYN, KA, QA	Plasma	HPLC	4,25	15	TRP$^{\#}$,KYN*, KA$^{\#}$, QA*
12	Erhardt, Lim et al. 2013	Sweden	MDD-Suicidal	Healthy Control	29	7	36	-	30(18-66)	KA, QA	CSF	HPLC-GCMS	4,5	15	KA*, QA$^{\#}$
13	Gabbay, Klein et al. 2010	USA	MDD-Melancholia	Healthy Control	20 9/11	22 9/13	42 18/24	15.6(2.0)	16.0(2.7)	TRP, KYN	Plasma	HPLC	6,25	9,5	TRP*, KYN*
			MDD-non Melancholia		30 14/16		52 23/29	16.3(2.1)							TRP#, KYN*
14	Hoekstra, Fekkes et al. 2006	Netherland	BD-Psychotic feature	Healthy Control	20 14/6	20 14/6	40 28/12	50,2(13,8)	50,1(13,5)	TRP	Plasma	HPLC	3,75	15	TRP*
15	Maes, Jacobs et al. 1990	Belgium	MDD-Melancholia	Healthy Control	15 6/9	16 8/8	31 14/17	41.0(15.8)	40.1(10.1)	TRP	Plasma	LC	6	10,5	TRP*
					22 10/12		38 18/20	43.9(13.8)							TRP*
					13 6/7		29 14/15	52.2(14.5)							TRP*
16	Maes, Meltzer et al. 1993	Belgium	MDD-Melancholia	Healthy Control	7	8	15	33.0(13.1)	44.1(13.1)	TRP	Plasma	LC	6,75	9,5	TRP*
					7	8	15	51.7(17.0)							TRP*
					10	8	18	54.8(12.5)							TRP*
17	Maes, De Backer et al. 1995	Belgium	MDD-Melancholia	Healthy Control	41 17/24	50 26/24	91 43/48	42.1(14.2)	40.7(11.3)	TRP	Plasma	LC	6,25	12	TRP*
					47 12/35		97 38/59	47.3(13.5)							TRP*
					35 14/21		85 40/45	51.7(15.0)							TRP*
18	Maes, Wauters et al. 1996	Belgium	MDD-Melancholia	Healthy Control	12 4/8	24 11/13	36 15/21	42,8(11,9)	41,1(13,5)	TRP	Serum	HPLC	5,75	11,5	TRP*
					21 3/18		45 14/31	48,7(14,3)							TRP*
					9 3/6		33 14/19	58,7(14)							TRP*
19	Milaneschi, Allers et al. 2021	Netherland	MDD-remitted-Melancholia	Healthy Control	753 226/527	642 246/396	1395 472/923	43,5(12,5)	41,1(14,7)	TRP,KYN, KA, QA	Plasma	-	3	17,5	TRP*,KYN*, KA*, QA*
			MDD-current-Melancholia		1100 358/743		1742 603/1139	40,8(12,1)							TRP$^{\#}$,KYN*, KA*, QA*

20	Miller, Llenos et al. 2006	USA	MDD-Psychotic feature	Healthy Control	14	14	28	45.9(9.7)	48.6(10.8)	KYN, KA	Brain Tissue	HPLC	4,75	12,5	KYN$^{\#}$, KA$^{\#}$
			BD-Psychotic feature		14	14	28	41.8(11.9)							KYN$^{\#}$, KA$^{\#}$
21	Møller 1993	Denmark	Depression-Melancholia	Healthy Control	26<br>8/18	55<br>16/39	81<br>24/57	52(10)	38(13)	TRP	Plasma	IEC	3,25	17	TRP*
22	Myint, Kim et al. 2007a	South Korea	BD-Psychotic feature	Healthy Control	39<br>15/24	80<br>40/40	119<br>55/64	37.6(11.6)	39.0(8.7)	TRP, KYN, KA, 3HA	Plasma	HPLC	6	12	TRP*, KYN*, KA*, 3HA$^{\#}$
23	Myint, Kim et al. 2007b	South Korea	Depression-Melancholia	Healthy Control	58<br>48/10	189<br>32/157	247<br>80/167	44.6(14.6)	32.4(10.6)	TRP, KYN, KA, 3HA	Plasma	HPLC	5,75	15	TRP*, KYN*, KA*, 3HA$^{\#}$
24	Pompili, Lionetto et al. 2019	Italy	MDD-Suicidal	Healthy Control	49<br>24/25	78<br>34/44	127<br>58/69	36,6(8,5)	40,4(12,3)	TRP, KYN AA, XA	Plasma	LCMS	3	18,5	TRP*, KYN AA, XA
25	Price, Charney et al. 1991	USA	Depression-Melancholia and psychotic feature	Healthy Control	126<br>38/88	58<br>17/41	184<br>58/126	43(14)	38(13)	TRP	Serum	HPLC	7	15	TRP*
26	Quintana 1992	Spain	MDD-Melancholia	Healthy Control	25<br>10/15	25<br>10/15	50<br>20/30	54(9,3)	-	TRP	Plasma	-	5,75	13,5	TRP*
27	Ryan, Allers et al. 2020	Ireland	Depression-Psychotic feature	Healthy Control	94<br>36/58	57<br>20/37	151<br>56/95	55.4(14.7)	51.7(11.4)	TRP, KYN KA, AA, 3HK, XA 3HA, QA PA	Plasma	LC/MS	6	13,5	TRP*, KYN* KA*, AA*, 3HK*, XA* 3HA*, QA* PA*
28	Savitz, Drevets et al. 2015d	USA	MDD-Suicidal	Healthy Control	53<br>11/42	47<br>18/29	100<br>29/71	34.6(9.8)	34.3(11.4)	TRP, KYN KA, 3HK, QA	Serum	LC–MS/MS	7	11,5	TRP*, KYN* KA*, 3HK$^{\#}$, QA$^{\#}$
29	Sellgren, Gracias et al. 2019	Sweden	BD-Psychotic feature	Healthy Control	163<br>64/99	114<br>52/62	277<br>116/161	34.2(3.3)	35.1(3.1)	KA	Plasma	HPLC	6	13,5	KA*
					94<br>39/55	113<br>51/62	207<br>90/117	36(20.5)	-		CSF				KA$^{\#}$
30	Song, Lin et al. 1998	Belgium	Depression-Melancholia	Healthy Control	6<br>2/4	14<br>6/8	20<br>8/12	50.3(15.3)	45.5(15.5)	TRP	Plasma	HPLC	4,25	13,5	TRP*
31	Steiner, Walter et al. 2011	Germany	Depression-Suicidal	Healthy Control	12<br>6/6	10<br>5/5	22<br>11/11	51(9)	56(6)	QA	Brain Tissue	ELISA	3,75	16	QA$^{\#}$
32	Sublette, Galfalvy et al. 2011	USA	MDD-Suicidal	Healthy Control	30<br>16/14	31<br>10/21	61<br>26/35	37.8(13.08	35.6(13.9)	TRP, KYN	Serum	HPLC	4	14	TRP*, KYN$^{\#}$
33	Trepci, Sellgren et al. 2021	Sweden	BD-Suicidal	Healthy Control	101<br>40/61	80<br>39/41	181<br>79/102	44.2(13.9)	34.7(12.4)	TRP, KYN KA, PA, QA	CSF	UPLC-MS/MS	7	16,5	TRP*, KYN$^{\#}$ KA$^{\#}$, PA$^{\#}$, QA$^{\#}$
34	Zhou, Zheng et al. 2018	China	MDD-Suicidal	Healthy Control	84<br>39/45	60<br>35/25	144<br>74/70	35.0(12.1)	31.6(10.7)	TRP,KYN, KA	Serum	HPLC-MS/MS	5	12,5	TRP*,KYN*, KA*

35	Messaoud, Mensi et al. 2019	Tunis	MDD	Healthy Control	56 20/36	40 15/25	96 35/61	34.2(11.3)	38.7(9.2)	TRP, KYN	Plasma	ECLIA	6	10	TRP*, KYN#

*: Indicates that patients have reduced level of the measured metabolite compared to healthy control

#: Indicates that patients have increased level of the measured metabolites compared to healthy control

TRP: Tryptophan, KYN: Kynurenine, KA: Kynurenic acid, 3HK: 3-Hydroxykynurenine, AA: Anthranilic acid, 3HA: 3-Hydroxyanthranilic acid, XA: Xanthurenic acid, QA: Quinolinic acid, PA: Picolinic acid, MDD: Major depressive disorder, BD: Bipolar disorder, MDD-M: Major depressive disorder with melancholia, DD: Dysthymic disorder, UD: Unipolar depression, HPLC: High performance liquid chromatography, HPLC-MS/MS: High performance liquid chromatography with tandem mass spectrometry, CE-TOFMS: Capillary Electrophoresis Time Of Flight Mass Spectrometer, LC-MS/MS: Liquid chromatography with tandem mass spectrometry, UPLC-MS/MS: Ultra performance liquid chromatography with tandem mass spectrometry, LCECA: Liquid, chromatography electrochemical array, XLC-MS/MS: Extraction-liquid chromatographic-tandem mass spectrometric, GCMS: Gas chromatography–mass spectrometry, LC: Liquid chromatography, HPLC-UV: High perfomance liquid chromatography- Ultra-violate

Table S5. Results of Meta-regression

Variables	No. of Studies	Covariates	1-sided p-value	Z-Value
TRP	29	Plasma	0.030	-1.88
TRP/CAAs	7	ECT-No	0.001	-3.10
CAAs	5	Sample size	0.042	1.72
KYN	17	Plasma	0.007	-2.44
KA/KYN	9	Male gender	0.048	-1.66
		Female gender	0.038	1.77
(KYN+3HK+3HA+XA+QA+PA)	26	Plasma	0.003	-2.75
KA	13	Latitude	0.015	2.16
		Plasma	0.026	-1.94
	12	Partially Medicated	0.049	-1.65
QA	14	Latitude	0.004	2.64
		Plasma	0.014	-2.17
	13	Partially Medicated	0.000	4.76

TRP: Tryptophan, KYN: Kynurenine, KA: Kynurenic acid, 3HK: 3-Hydroxykynurenine, AA: Anthranilic acid, 3HA: 3-Hydroxyanthranilic acid, XA: Xanthurenic acid, QA: Quinolinic acid, PA: Picolinic acid, MDD: CAAs: Competing amino acids.

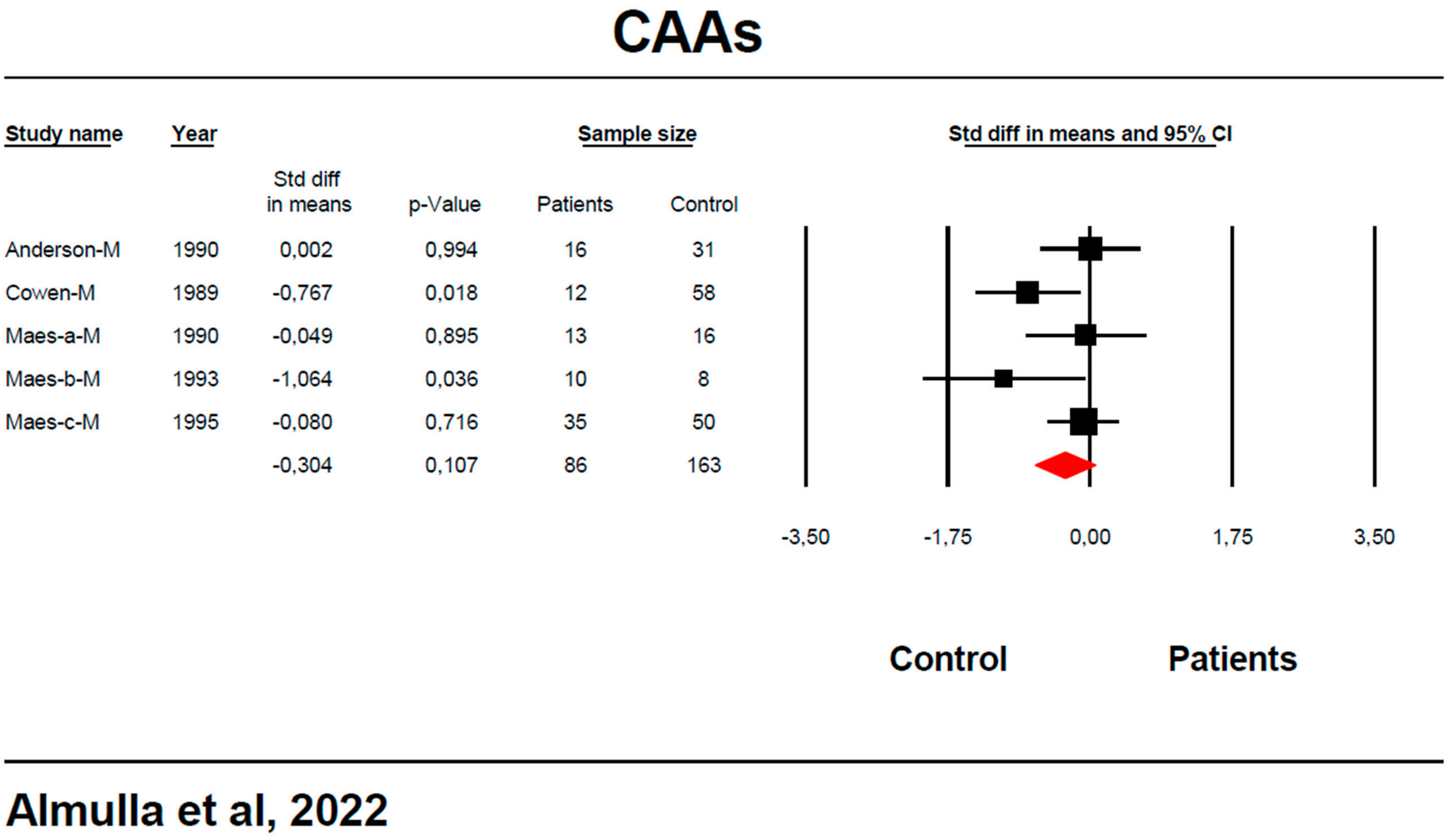

Figure S1: The forest plot of competing amino acids (CAAs) level between severe affective disorder patients and healthy control.

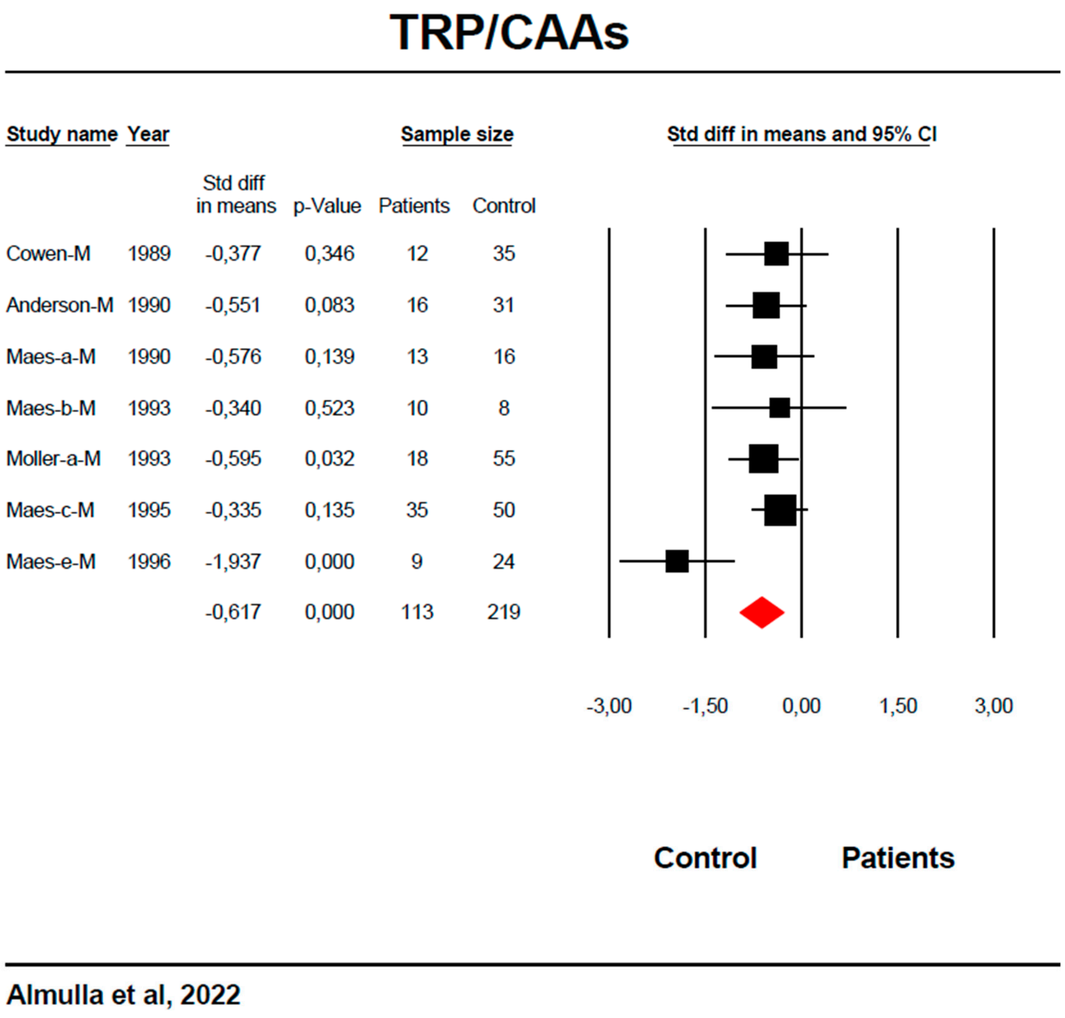

Figure S2: The forest plot of TRP/CAAs ratio between severe affective disorder patients and healthy control.

KYN

Study name	Year	Std diff in means	p-Value	Sample size: Patients	Sample size: Control
Aarsland-P	2019	0,320	0,334	27	14
Achtyes-S	2020	-0,442	0,009	87	60
Ameele-P	2020	-0,448	0,067	34	35
Bradley-S	2015	-0,662	0,037	20	22
Clark-S	2016	-0,119	0,594	45	36
Dahl-M	2015	-0,281	0,237	38	34
Gabbay-M	2010	-0,224	0,469	20	22
Milaneschi-M	2021	-0,200	0,000	927	642
Myint-a-P	2007	-0,302	0,123	39	80
Myint-b-S	2007	-0,129	0,389	58	189
Pompili-S	2018	-1,659	0,000	49	78
Ryan-P	2020	-0,089	0,596	94	57
Savitz-c-S	2015	0,044	0,846	35	58
Sublette-S	2011	0,883	0,008	14	31
Trepci-S	2021	0,739	0,000	101	80
Zhou-a-S	2018	-0,045	0,790	84	60
Messaoud-S	2018	0,813	0,000	56	40
		-0,114	0,350	1727	1538

Std diff in means and 95% CI

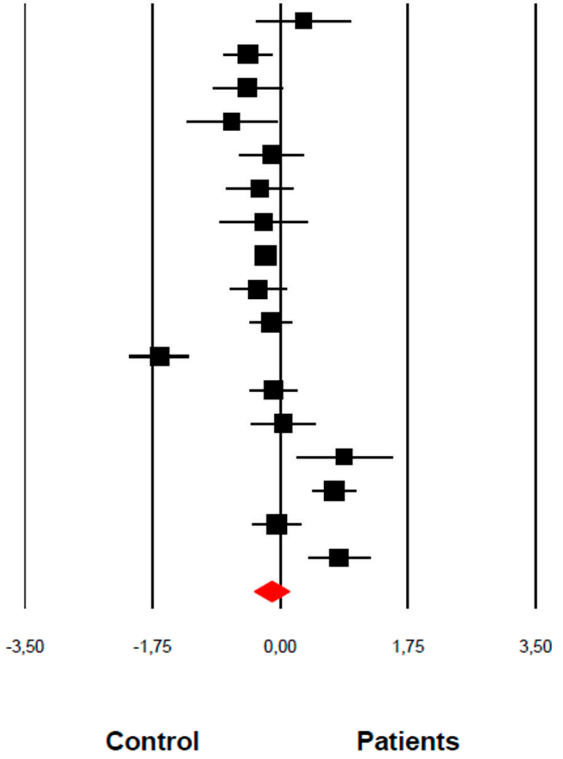

Almulla et al, 2022

Figure S3: The forest plot of kynurenine (KYN) level between severe affective disorder patients and healthy control.

(KYN+3HK+3HA+XA+QA+PA)

Study name	Year	Std diff in means	p-Value	Sample size: Patients	Sample size: Control
Aarsland-P	2019	-0,299	0,371	27	14
Myint-a-P	2007	-0,103	0,600	39	80
Myint-b-S	2007	-0,041	0,785	58	189
Ryan-P	2020	-0,266	0,118	94	57
Bradley-S	2015	-0,667	0,036	20	22
Gabbay-M	2010	-0,256	0,410	20	22
Ameele-P	2020	-0,448	0,067	34	35
Clark-S	2016	-0,546	0,018	45	36
Savitz-c-S	2015	0,069	0,761	35	58
Pompili-S	2018	-0,920	0,000	49	78
Achtyes-S	2020	-0,402	0,018	87	60
Bay-Richte-S	2015	3,244	0,000	30	36
Dahl-M	2015	-0,189	0,424	38	34
Erhardt-S	2013	0,967	0,000	31	36
Miller-P	2006	0,253	0,505	14	14
Miller-P	2006	0,187	0,621	14	14
Trepci-S	2021	0,539	0,000	101	80
Zhou-a-S	2018	-0,045	0,790	84	60
Milaneschi-M	2021	-0,131	0,011	927	642
Sublette-S	2011	0,883	0,008	14	31
Brundin-CeS	2016	-2,611	0,000	12	36
Brundin-PeS	2016	-0,223	0,445	21	29
Brundin-PeS	2016	-0,350	0,490	7	29
Busse-S	2015	1,459	0,012	6	10
Steiner-S	2011	0,828	0,067	12	10
Messaoud-S	2018	0,813	0,000	56	40
		0,048	0,692	1874	1752

Std diff in means and 95% CI

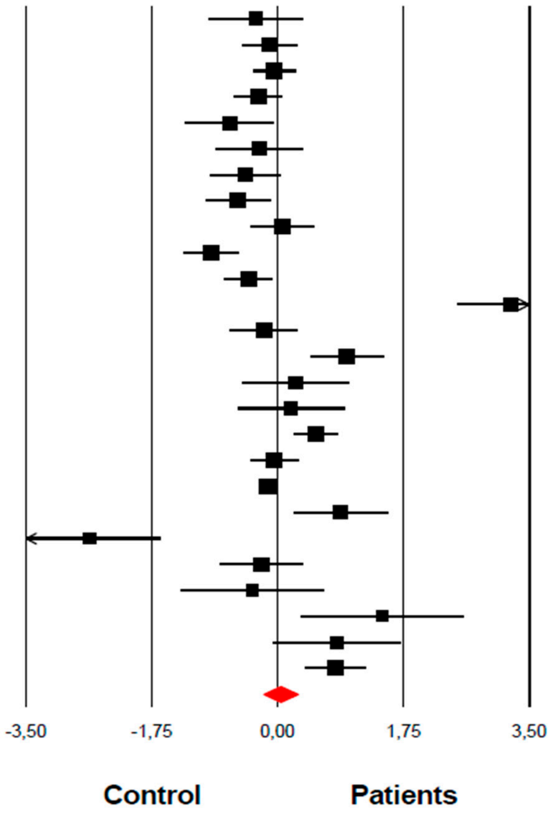

Almulla et al, 2022

Figure S4: The forest plot of neurotoxic composite (KYN+3HK+3HA+XA+QA+PA) level between severe affective disorder patients and healthy control.

KA/KYN

Study name	Year	Subgroup within study	Statistics for each study: Std diff in means	p-Value	Sample size: Patients	Control
Dahl-M	2015	Melancholic	0,196	0,408	38	34
Milaneschi-M	2021	Melancholic	0,042	0,417	927	642
		Melancholic	0,049	0,333	965	676
Aarsland-P	2019	Psychotic	-0,399	0,230	27	14
Miller-P	2006	Psychotic	-0,063	0,867	14	14
Miller-P	2006	Psychotic	-0,063	0,868	14	14
Myint-a-P	2007	Psychotic	-0,123	0,533	39	80
Ryan-P	2020	Psychotic	-0,264	0,121	94	57
		Psychotic	-0,201	0,066	188	179
Achtyes-S	2020	Suicidal	0,061	0,718	87	60
Clark-S	2016	Suicidal	-0,153	0,496	45	36
Messaoud-S	2018	Suicidal	-0,813	0,000	56	40
Myint-b-S	2007	Suicidal	-0,407	0,008	58	189
Savitz-c-S	2015	Suicidal	-0,174	0,443	35	58
Trepci-S	2021	Suicidal	-0,070	0,649	101	80
Zhou-a-S	2018	Suicidal	-0,147	0,385	84	60
		Suicidal	-0,231	0,024	466	523
		Overall	-0,035	0,410	1619	1378

Std diff in means and 95% CI

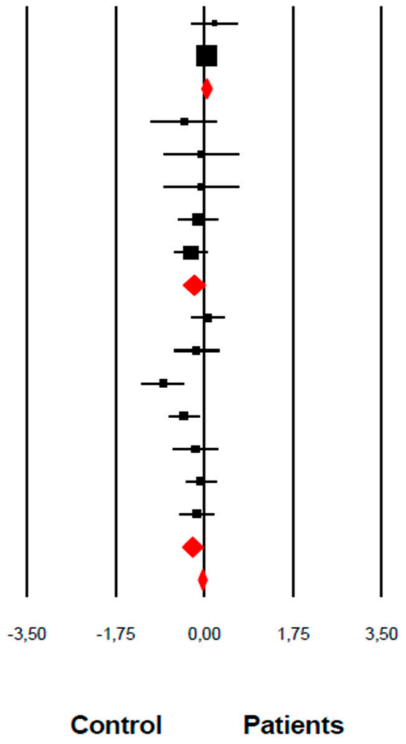

Almulla et al, 2022

Figure S5: The forest plot of kynurenic acid (KA)/kynurenine (KYN) ratio between severe affective disorder patients and healthy control.

KA

Study name		Std diff in means	p-Value	Patients	Control
Aarsland-P	2019	-0,478	0,152	27	14
Achtyes-S	2020	-0,319	0,059	87	60
Clark-S	2016	-0,425	0,060	45	36
Dahl-M	2015	0,111	0,640	38	34
Milaneschi-M	2021	-0,116	0,025	927	642
Miller-P	2006	0,126	0,739	14	14
Miller-P	2006	0,062	0,870	14	14
Myint-a-P	2007	-0,548	0,006	39	80
Myint-b-S	2007	-0,943	0,000	58	189
Ryan-P	2020	-0,616	0,000	94	57
Savitz-c-S	2015	-0,304	0,181	35	58
Trepci-S	2021	0,599	0,000	101	80
Zhou-a-S	2018	-0,339	0,046	84	60
		-0,260	0,024	1563	1338

(Sample size: Patients, Control)

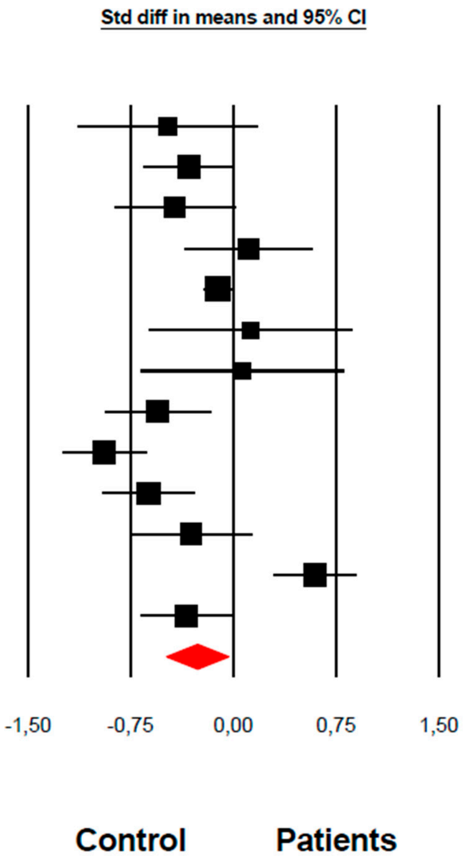

Almulla et al, 2022

Figure S6: The forest plot of kynurenic acid (KA) level between severe affective disorder patients and healthy control.

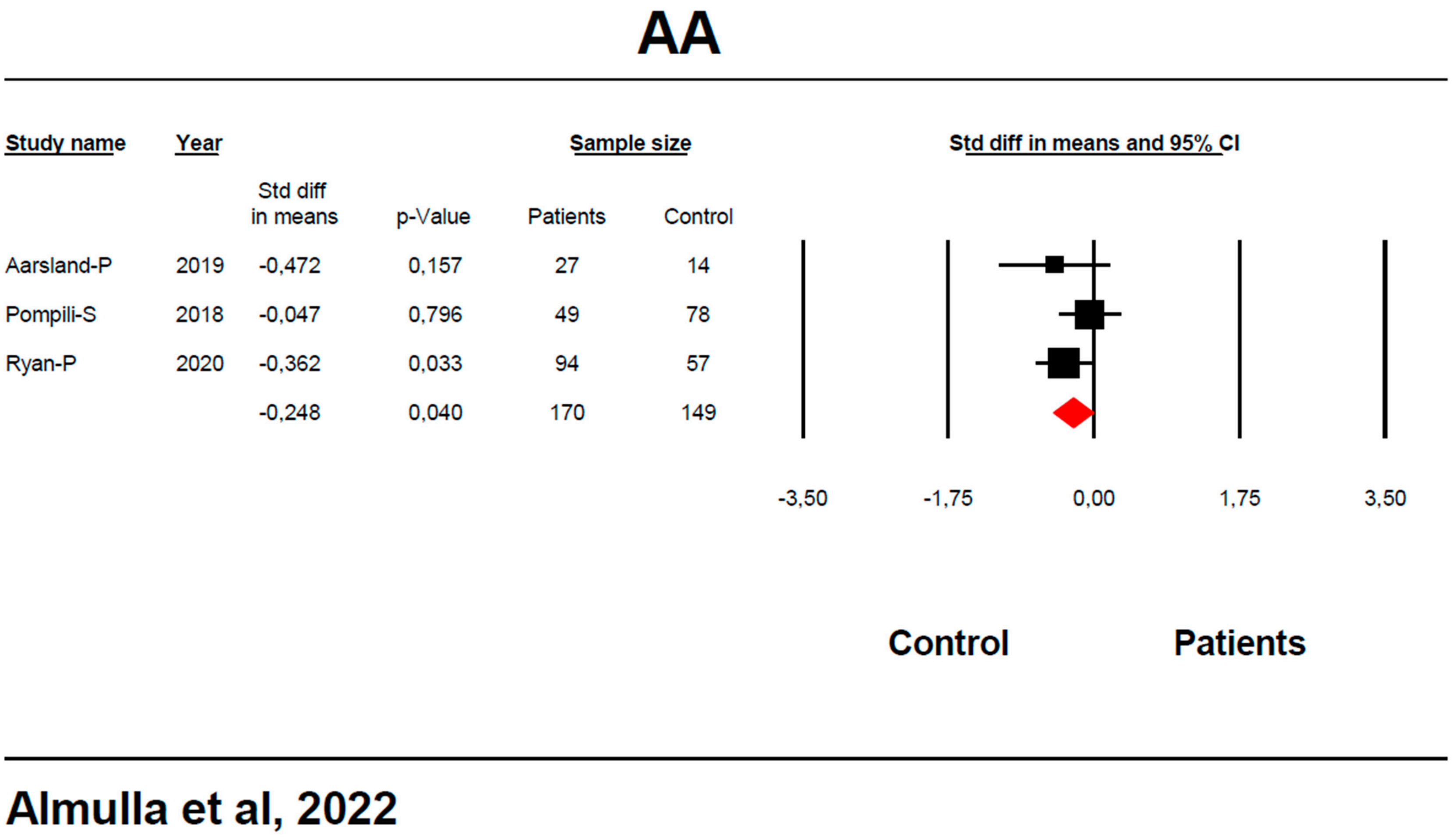

Figure S7: The forest plot of anthranilic acid (AA) level between severe affective disorder patients and healthy control.

QA

Study name	Year	Std diff in means	p-Value	Sample size: Patients	Sample size: Control
Steiner-S	2011	0,828	0,067	12	10
Erhardt-S	2013	0,967	0,000	31	36
Savitz-c-S	2015	0,204	0,369	35	58
Bay-Richte-S	2015	3,244	0,000	30	36
Dahl-M	2015	-0,098	0,679	38	34
Busse-S	2015	1,459	0,012	6	10
Clark-S	2016	-1,084	0,000	45	36
Brundin-PeS	2016	0,270	0,348	21	29
Brundin-PeS	2016	0,097	0,847	7	29
Aarsland-P	2019	0,077	0,815	27	14
Ryan-P	2020	0,174	0,301	94	57
Achtyes-S	2020	-0,363	0,032	87	60
Trepci-S	2021	0,402	0,008	101	80
Milaneschi-M	2021	-0,062	0,230	927	642
		0,358	0,041	1461	1131

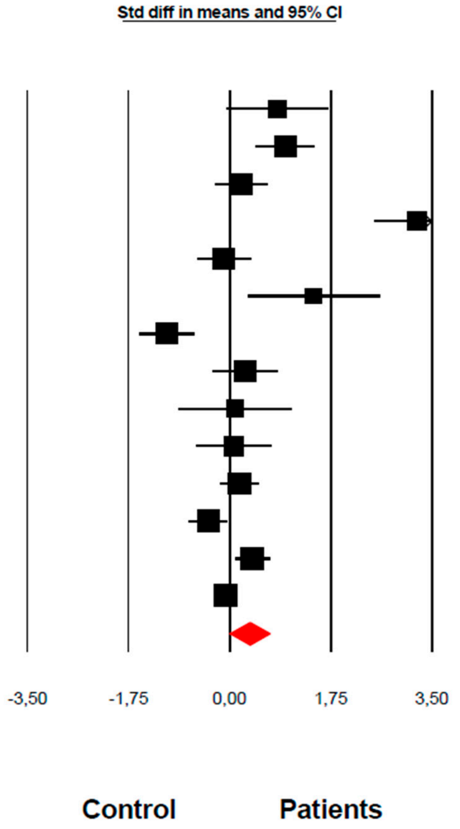

Almulla et al, 2022

Figure S8: The forest plot of quinolinic acid (QA) level between severe affective disorder patients and healthy control.

Abbreviations:

TRP: Tryptophan

KYN: Kynurenine

KA: Kynurenic acid

3HK: 3-Hydroxykynurenine

AA: Anthranilic acid

3HA: 3-Hydroxyanthranilic acid

XA: Xanthurenic acid

QA: Quinolinic acid

PA: Picolinic acid

CAAs: Competing amino acids

IDO: Indoleamine 2,3 dioxygenase

TDO: Tryptophan 2,3 dioxygenase

KAT: Kynurenine aminotransferase

KMO: Kynurenine 3-monooxygenase

KYNU: Kynureninase

NAD^+: Nicotinamide adenine dinucleotide

TRYCATs: Tryptophan Catabolites

TRYCAT pathway: Tryptophan catabolite pathway

SMD: Standardized mean difference

IL: Interleukin

IFN-γ: Interferon-gamma

O&NS: Oxidative and nitrosative stress

NAD: Nicotinamide adenine dinucleotide

MOOSE: Meta-Analyses of Observational Studies in Epidemiology

CSF: Cerebrospinal fluid

SD: Standard deviation

IOR: Interquartile range

ICS: Immune confounder scale

CI: Confidence intervals

LC-MS: Liquid chromatography-mass spectrometry

LC-MS/MS: Liquid chromatography with two mass spectrometry

UHPLC-MS: Ultra-high-performance liquid-chromatography- mass spectrometry

LC-HRMS: Liquid chromatography–high-resolution mass spectrometry

LC-UV: Liquid chromatography-UV detection

NMDA: N-methyl-D-aspartate

ELICA: Electrochemiluminescence immunoassay
